# Evaluation of the Diagnostic Performance and Validation of an AI-Assisted Fluorescence Imaging Device for Fecal Egg Counts Against the Manual McMaster Reference Method in Kiko Male Goats

**DOI:** 10.3390/ani16020248

**Published:** 2026-01-14

**Authors:** Ahmadreza Mirzaei, Alireza Rahmani Shahraki, Fiona P. Maunsell, Brittany N. Diehl

**Affiliations:** Department of Large Animal Clinical Sciences, University of Florida, Gainesville, FL 32611, USA; ahmadrez.mirzaei@ufl.edu (A.M.); alirezarahmanish@ufl.edu (A.R.S.); maunsellf@ufl.edu (F.P.M.)

**Keywords:** fecal egg count, artificial intelligence, goat parasitology, automated imaging system, diagnostic performance

## Abstract

Gastrointestinal parasites are a major health and economic concern in small ruminants. The classic microscopic approach using the manual McMaster method serves to quantitatively count parasite eggs, which are labor-intensive and prone to variation. Artificial intelligence-based systems (Parasight^®^, powered by Fecalsight AI™) could provide quicker and more objective alternatives; therefore, independent validation is necessary before clinical implementation. The objective of this study was to evaluate the agreement, classification consistency, and diagnostic performance of Parasight^®^ relative to the manual McMaster method, with a focus on its suitability as a screening and decision-support tool. Fecal samples from 44 Kiko goats over 3 sampling times were analyzed using both methods, with manual counts performed independently by 2 observers. Agreement between methods was assessed using Lin’s concordance correlation coefficient, Bland–Altman analysis, and Cohen’s Kappa for categorical classification. Diagnostic performance for identifying animals exceeding the clinical treatment threshold (>1000 eggs per gram) was evaluated using receiver operating characteristic (ROC) analysis, and regression modeling was used to characterize associations between methods. Manual observers showed high reliability, confirming the suitability of the McMaster method as a reference. Compared with manual counts, Parasight^®^ consistently underestimated egg counts, resulting in poor-to-moderate absolute agreement; however, it reliably ranked animals by parasite burden and showed excellent discrimination for identifying animals above the treatment threshold (AUC = 0.90–0.96). Regression analyses further demonstrated linear or curvilinear associations depending on egg counts. Overall, the Parasight^®^ device reliably captured relative parasite burden but required a lower operational threshold to match manual treatment decisions.

## Simple Summary

Gastrointestinal parasites are a major concern for goats and other grazing animals, resulting in poor growth, anemia, and in severe cases even death. Farmers have traditionally measured parasite levels by counting parasite eggs in feces under a microscope, but this procedure is slow, requires training, and is susceptible to individual variation. New automated technologies that use artificial intelligence have been developed to make this procedure faster and more consistent, but they need to be thoroughly evaluated before they can be adopted in daily practice. In this study, fecal parasite egg counts from male goats maintained under identical management conditions were used to compare an artificial intelligence–based egg-counting technique with the conventional microscopy method. The agreement between the two approaches, the accuracy of the automated device in identifying animals requiring treatment, and diagnostic performance of the automated system were assessed. The automated system successfully ranked animals by level of infection and showed excellent ability to detect animals above the treatment threshold, but it consistently counted fewer eggs than the manual method. This means the device can be useful, but the treatment threshold must be adjusted. With proper calibration, this technology could improve parasite control and reduce drug resistance.

## 1. Introduction

Internal parasites pose a significant concern for both people and animals [1]. In small ruminants, gastrointestinal parasitism may result in substantial direct and indirect economic losses; for example, a 16% reduction in farmers’ profit, primarily due to a 50% reduction in weight gain, has been referred to parasitic infections in goats [2]. Parasitism is also associated with anorexia, decreased resilience to illness, and in severe cases, mortality [3]. The effects of parasitism on the economy and small ruminants’ health are exacerbated by the emerging challenge of increase in anthelmintic resistance [4]. Therefore, both the World Health Organization and the World Association for the Advancement of Veterinary Parasitology have recommended fecal egg counts (**FECs**) as a monitoring tool in humans [1] and for assessing intestinal parasite infections in livestock [5].

An essential component of targeted selective treatment programs in small ruminants is FEC [6] and it can also be used to monitor drug efficacy and guide treatment strategies based on parasite species prevalence and resistance status [7]. Despite its widespread use, FEC methods are time consuming, require laboratory equipment and trained personnel, meanwhile the underlying methodology has not altered much in the last century [8,9].

The McMaster method, first introduced in the 1930s [9], is a traditional quantitative egg-counting chamber technique that is still widely accepted as the industry standard today. Over time, multiple adaptations of the McMaster technique have been developed, including chamber-based methods such as the Moredun method [10], FECPAK [11], FLOTAC [12], and Mini-FLOTAC [13], all of which can be considered enhancements of the original McMaster principle. Recently, an alternative approach of quantifying fecal eggs has been introduced by implementing new technologies that combine dual-step sample processing with fluorescence-based imaging and artificial intelligence-driven classification and counting (Parasight^®^; MEP Equine Solutions, Lexington, KY, USA). This approach is consistent with earlier methods that used fluorescent chitin-binding probes to label helminth eggs, combined with automated image analysis for detection and enumeration [14,15]. Although the proprietary details of the commercial platform remain undisclosed, the system appears to rely on the same fluorescence-imaging-plus-AI framework. This is an important shift from both manual microscopy and traditional McMaster flotation approach to implementation of sophisticated and objective image-recognition systems that employ fluorescence associated techniques.

Adoption of novel diagnostic technologies is intrinsically challenging, as all procedures regardless of design might be subjected to variability arising from both random and systematic sources of error. Understanding this variability and rigorously validating new methods is therefore essential before clinical implementation. The primary objective of the present study was to evaluate the agreement and classification consistency of an AI-assisted fecal egg counting device (Parasight^®^) relative to the conventional McMaster technique in Kiko male goats, with the aim of assessing its suitability as a screening and clinical decision-support tool. In addition, we evaluated the diagnostic performance of the Parasight^®^ system for identifying animals exceeding the commonly applied therapeutic threshold for Trichostrongylus infection (≥1000 eggs per gram). Finally, we examined the relationship between Parasight^®^ and manual egg counts across multiple sampling times to determine how effectively the automated system captured relative changes in fecal egg burden over time.

## 2. Materials and Methods

### 2.1. Animals and Husbandry

A total of 68 male Kiko goats of comparable age were enrolled in the annual University of Florida Buck Test program. At enrollment, the goats had a mean body weight of 25.6 ± 0.51 kg (range: 18.1–34.9 kg), an average body condition score (**BCS**) of 2.9 ± 0.01 (range: 2.5–3.5), and a mean FAMACHA© score of 1.4 ± 0.01 (range: 1–2). The animals were sourced from multiple external farms and transported to the University of Florida in June 2025, where they were maintained for three months at the Institute of Food and Agricultural Sciences (**IFAS**) small ruminant research facility. All goats were group-housed on outdoor pastures under standard husbandry practices, with unrestricted access to Bahiagrass pasture and water. Upon arrival, combination oral anthelmintic treatments (Levamisole 12 mg/kg, Fenbendazole 10 mg/kg, Cydectin 0.4 mg/kg) were administered on day 0 of the test. No anthelmintic treatment was administered during the study period, allowing the natural progression of gastrointestinal nematode infection.

Over the course of the program, each animal underwent seven rounds of FEC evaluation. For the present study, only the final three FECs were included, ensuring that all animals were maintained under identical environmental and management conditions for at least 42 days before the first measurement.

All goats underwent routine bi-weekly health monitoring by University of Florida, College of Veterinary Medicine veterinarians, which included physical examinations, measurement of BCS and body weight, FAMACHA© scoring, measurement of scrotal circumference and fecal sampling for FECs. All goats were maintained under uniform management conditions at the IFAS facility throughout this experiment.

### 2.2. Animal Selection and Sampling

From the initial population of 68 male Kiko goats enrolled in the University of Florida Buck Test (average age at the start of the program = 143.14 ± 5.2 days), 44 animals were randomly selected for inclusion into this study. Goats were eligible if they were clinically healthy, within the target age range, and maintained under uniform management and nutrition throughout the test period. Animals had not received any anthelmintic or antimicrobial treatment during the test period and showed no evidence of systemic illness, diarrhea, or other clinical abnormalities. Only goats providing a sufficient quantity of fresh feces for analysis, where traceable through individual identification (ear tag), were included. To account for natural variation in body weight, goats were stratified into quartiles (Q1–Q4) based on baseline weights, and 11 goats were randomly chosen from each quartile using a random number generator (Microsoft Excel). The mean (±SEM) body weight in each quartile was: Q1 = 20.23 ± 0.56 kg, Q2 = 24.63 ± 0.25 kg, Q3 = 27.67 ± 0.27 kg, and Q4 = 30.61 ± 0.47 kg. Unique animal IDs and block assignments were recorded. This block-randomization approach ensured a balanced representation across body weight categories and minimized potential bias related to body weight. Due to the lack of fecal sample collection at specific time points, 8, 4, and 12 samples were excluded from the first, second, and third sampling periods, respectively.

Each goat was sampled once every other week for a total of three consecutive time points (T1, T2 and T3), yielding three sampling events per animal. At each time point, a single fecal sample was collected and then divided into two subsamples. One subsample was analyzed using the Parasight^®^ automated egg-counting device, while the other was examined manually by two independent observers using the McMaster technique. Manual observers were blinded to both the Parasight^®^ results and to each other’s counts to minimize observer’s bias. Manual fecal egg counts were performed independently by two licensed veterinarians, each with approximately 10 years of experience in clinical parasitology and laboratory-based diagnostic microscopy. Both observers had extensive prior experience with the McMaster technique in routine clinical and research settings. In addition, they received similar formal training in parasitological diagnostics, as they completed their veterinary education together and subsequently underwent comparable postgraduate training. This shared educational and professional background ensured consistency in egg identification and counting procedures and strengthened the reliability of the manual McMaster method used as the reference standard in this study.

Immediately after collection, fecal samples were placed in a cooler (≈4 °C) and subsequently stored under refrigeration until analysis. All manual McMaster counts were completed within 6 days of collection, with samples maintained at 4 °C throughout the storage period. This time window was chosen because refrigerated fecal egg counts are reported to remain relatively stable for approximately one week, whereas detectability begins to decline when refrigeration extends beyond ~8 days [16]. Additionally, both manual observers read slides concurrently to avoid differences in slide quality or sample condition due to processing time.

All fecal egg counts were expressed as eggs per gram (**EPG**) of feces. In addition, technicians responsible for sample handling were blinded to sample identity to ensure objectivity throughout the study.

### 2.3. Egg Counting Methods

**Figure 1:**
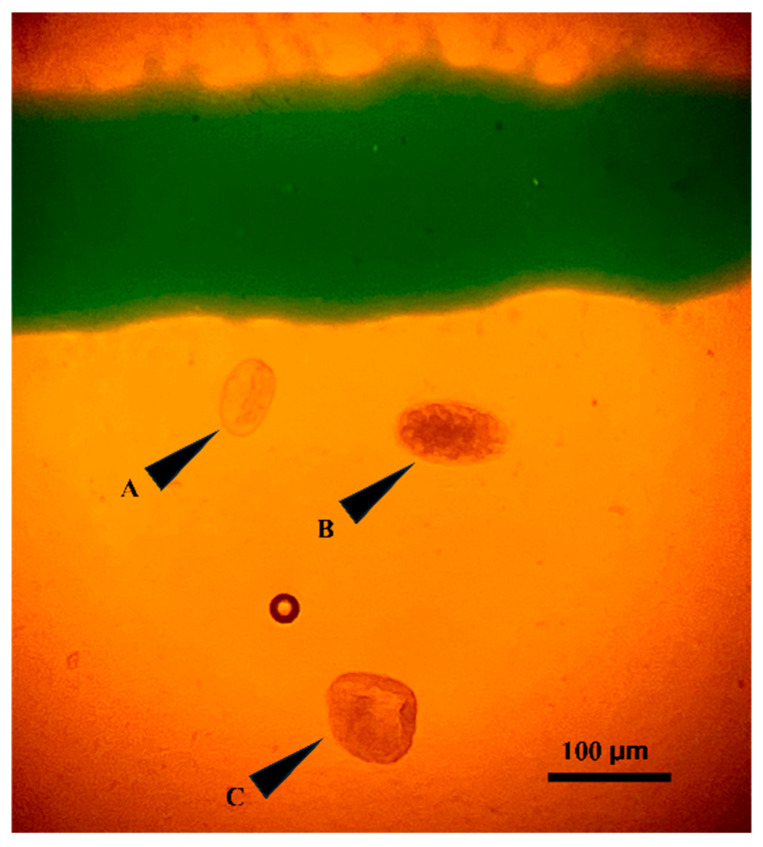
Representative micrograph showing the three main gastrointestinal parasite eggs identified in Kiko goats using the McMaster technique. From top to bottom: (A) Strongyloides (elliptical, larvated egg), (B) Trichostrongylus (oval, thin-shelled egg with segmented contents), and (C) Moniezia (triangular or irregular-shaped egg). The image was captured under bright-field microscopy using a McMaster chamber.

Each fecal sample was divided into two equal portions: one designated for manual McMaster counting and the other for analysis using Parasight^®^ system (Parasight System Inc., Lexington, KY, USA). From the portion assigned to the manual McMaster method, two replicate slides were prepared and examined independently to account for within-sample variability and ensure counting precision. Fecal egg counts were performed using the modified McMaster technique as previously described [17]. Briefly, approximately 2 g of fresh feces was homogenized with 28 mL of saturated salt flotation solution (specific gravity ~1.20). The suspension was strained through a tea strainer to remove large debris, and each chamber of a McMaster slide (0.15 mL per chamber) was carefully filled with filtration. Slides were allowed to stand for ~5 min to permit egg flotation and then examined under a compound microscope at 100× magnification. Strongyle-type eggs (*Haemonchus contortus*, *Ostertagia* spp., *Trichostrongylus* spp., *Cooperia* sp.), *Moniezia* spp. and *Strongyloides* spp. were counted by the two observers within the ruled grid areas of both chambers to obtain fecal egg counts. Figure 1. Representative micrographs of an example of the three nematode egg types identified in goat fecal samples. The total number of eggs was multiplied by 50 to obtain eggs per gram. To ensure consistency, all slides were read within 60 min of preparation, and fecal samples were stored at 4 °C and processed within 6 days of collection. The sensitivity of the method was 50 EPG.

Automated FECs were performed with a commercial system (Parasight^®^ powered by FecalsightAI™; MEP Equine Solutions, Lexington, KY, USA) which concentrates eggs by filtration, fluorescently labels them, and enumerates them using automated imaging and a deep-learning classifier, following the fluorescence-based approach described by [15]. Detailed proprietary implementation is not publicly disclosed.

### 2.4. Sample Size Calculation

To evaluate the agreement between the Parasight^®^ automated egg-counting device and the manual McMaster method, the concordance correlation coefficient (CCC) method was implemented and sample size was determined using the Lin’s method [18] which estimates the number of subjects needed to detect a meaningful difference between an expected CCC and a null value at a specified significance level and power. We assumed an expected CCC of 0.80, representing good agreement based on conventional thresholds, and a minimum acceptable (null) value of 0.60. With a two-sided alpha of 0.05 and 80% power, the required sample size was 40 animals. To account for potential sample loss, we increased enrollment by 10%, resulting in a final study population of 44 animals. Fecal egg shedding in small ruminants can fluctuate over time due to environmental factors, and physiological stressors, therefore we incorporated three sampling times spaced two weeks apart to improve the reliability of our evaluation. Multiple sampling timepoints allowed us to assess the temporal consistency of agreement between methods and to capture potential differences in method performance across varying parasite burdens. This design also ensured that both the manual and automated methods were evaluated under conditions of low, moderate, and high egg counts, thereby providing a more comprehensive assessment of agreement across the full biologically relevant range of fecal egg shedding.

### 2.5. Statistical Analysis

All statistical analyses were performed using R statistical software (version 4.3.2; R Foundation for Statistical Computing, Vienna, Austria). To test consistency among manual observers, the intraclass correlation coefficient (**ICC**) measurement was used for each of the samples in three sampling times separately for each parasite egg related to *Trichostrongylus*, *Moniezia*, *Strongyloides* and Total EPG is the sum of *Trichostrongyle* spp., *Strongyloides* and *Moniezia*. A two-way random-effects model with absolute agreement was applied, and ICC values were reported with 95% confidence intervals. Interpretation followed standard benchmarks that includes values < 0.50 = poor, 0.50–0.75 = moderate, 0.75–0.90 = good, and >0.90 = excellent reliability [19]. Agreement between observers was further evaluated using Bland–Altman analysis, reporting mean bias and 95% limits of agreement.

To compare the Parasight^®^ automated device with manual McMaster reference, the reference value was calculated as the average of the two observers’ counts for each sample and time point. This agreement was assessed for *Trichostrongyle*, *Strongyloides* and Total EPG (the current configuration of the Parasight^®^ device is limited to detecting and counting *Trichostrongylus* and *Strongyloides* eggs only) which is sum of *Trichostrongyle* and *Strongyloides* using Lin’s concordance correlation coefficient. To address the right-skewed distribution typically observed in fecal egg count data, log transformation was initially explored during preliminary analyses; however, agreement statistics presented in the final tables were computed on the original EPG scale to allow direct interpretation of absolute differences between methods. Point estimates and 95% confidence intervals for CCC were obtained using the epi.ccc() function (epiR package, version 4.3.2), and the bias correction factor (C*_b_*) was reported to quantify scale and location shift. Spearman’s rank correlation coefficient was additionally computed to assess monotonic association.

Bland–Altman analyses were performed on the original EPG scale to quantify absolute disagreement between Parasight^®^ and manual McMaster counts. In this framework, mean bias represents the average difference in EPG (Parasight^®^—manual), and the limits of agreement (LoA) describe the range within which most measurement differences are expected to fall. This approach provides a direct interpretation of numerical under- or overestimation in biologically meaningful units. Initial exploratory analyses was performed on log-transformed data to evaluate proportional disagreement. In order to allow for comparison with Table 1 and to maintain consistency with the manual method’s measurement scale, Table 2 represents the final results in the original EPG scale after back-transformation. Observer agreement on categorical classification of *Trichostrongylus* egg counts (>1000 vs. ≤1000 EPG) was assessed with Cohen’s κ, interpreted as: ≤0.20 = slight, 0.21–0.40 = fair, 0.41–0.60 = moderate, 0.61–0.80 = substantial, and ≥0.81 = almost perfect agreement [20]. For categorization of the continued measures of FECs of *Trichostrongylus*, we used the cut off 1000 EPG of parasite eggs to consider it as a clinical infection in goats. However, it is worth mentioning that no single universal threshold has been set for *Trichostrongylus* and not all the thresholds can apply to all situations. General guidelines suggest treating goats with FECs above 200 EPG with treatment becoming necessary for higher counts such as 500 EPG. However, the most important factor which determines the treatment is clinical signs of the disease which indicate the animals with high FEC, combined with clinical signs, to be targeted for clinical treatment.

The diagnostic performance of the Parasight^®^ device relative to the manual McMaster method was evaluated using receiver operating characteristic (**ROC**) analysis. The average classification from the two manual observers (>1000 vs. ≤1000 eggs per gram [EPG]) served as the reference standard, and Parasight^®^ counts were treated as the continuous predictor. An area under the curve (**AUC**) of 0.5 indicates a non-informative test (equivalent to random classification), whereas an AUC of 1.0 represents perfect discrimination. Test accuracy was interpreted as follows: 0.90–1.00 = excellent, 0.70–0.89 = acceptable, and <0.70 = poor [21]. The optimal Parasight^®^ cutoff value was determined by maximizing Youden’s J statistic (sensitivity + specificity − 1). Diagnostic performance was further evaluated at the clinical decision threshold of 1000 EPG, with sensitivity, specificity, positive predictive value (**PPV**), and negative predictive value (**NPV**) reported. All ROC analyses were conducted separately for each sampling time point (T1–T3).

Regression analyses were conducted separately for each sampling time to evaluate the association between Parasight^®^ total egg counts and the manually derived total egg counts (average of two observers at each time point). For each timepoint, linear and quadratic variables (Manual + Manual^2^) were initially fitted, and the quadratic term was retained only when its contribution was statistically significant (*p* ≤ 0.10).

## 3. Results

### 3.1. Inter-Observer Reliability and Agreement of Two Manual McMaster Egg Counts Across Sampling Times

**Figure 2:**
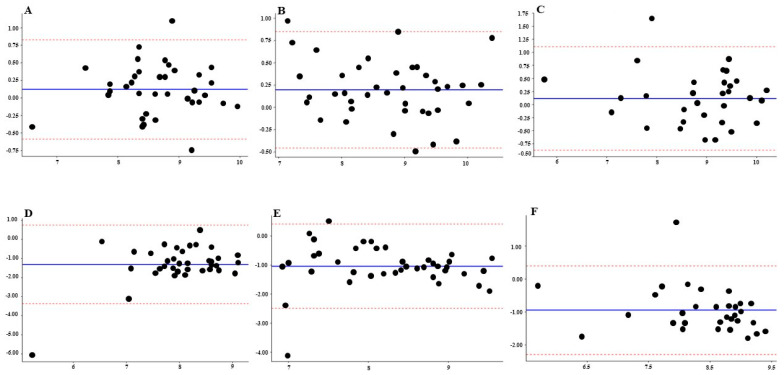
Bland–Altman plots comparing log-transformed fecal egg counts (FEC) between methods and observers across sampling times. Panels (**A**–**C**) represent inter-observer reliability and agreement between two manual McMaster counts at 1st, 2nd and 3rd sampling times, respectively. Panels (**D**–**F**) represent agreement between the Parasight^®^ automated system, and the average of two manual McMaster counts at 1st, 2nd and 3rd sampling times respectively. The x-axis shows the average of paired log-transformed FECs, and the y-axis shows the difference between paired measurements. The solid blue line indicates the mean bias (systematic difference) between methods or observers, while the dotted red lines represent the 95% limits of agreement.

The inter-observer reliability of manual McMaster counts was generally good to excellent across most parasite groups and sampling times (Table 1). At T1 sampling time, ICC values ranged from 0.64 (95% CI: 0.40–0.80) for *Strongyloides* (moderate agreement) to 0.93 (95% CI: 0.87–0.96) for *Trichostrongyle* counts (excellent agreement). Reliability for *Moniezia* was also high (ICC = 0.87). Bland–Altman analysis showed small mean biases (+61 EPG for *Trichostrongyle* and −215 EPG for *Moniezia*), though limits of agreement were wide for *Strongyloides* (−3218 to +4593 EPG). At T2 sampling time, agreement remained good, with ICC values between 0.80 (*Total EPG*) and 0.89 (*Moniezia*). Mean bias values were small (−65 EPG for *Moniezia*), but inter-observer differences reached over 1200 EPG for *Strongyloides*. At T3 sampling time, reliability was highest for *Moniezia* (ICC = 0.97, 95% CI: 0.94–0.99), and good for both *Trichostrongyle* (ICC = 0.85) and *Strongyloides* (ICC = 0.81). As expected, Total EPG counts representing the sum of *Trichostrongyle* and *Strongyloides* eggs showed good reliability (ICC = 0.79–0.84 across timepoints). Bland–Altman analysis revealed a high degree of consistency between the two manual McMaster observers across all sampling times (Figure 2A–C). The mean bias between observers was close to zero at each time point, indicating minimal systematic differences. The limits of agreement were relatively narrow, suggesting acceptable repeatability of manual counting under field conditions. However, greater dispersion of differences was noted at higher FECs values, consistent with increased variability at elevated parasite burdens. Overall, these findings confirm good inter-observer reliability of manual McMaster counts, although precision slightly decreased with increasing egg concentration.

### 3.2. Agreement Between Parasight^®^ and Average of Manual McMaster Egg Counts Across Sampling Times

Agreement between the Parasight^®^ automated device and the average manual McMaster counts from two observers ranged from poor to moderate across sampling times (Table 2). Lin’s CCC varied between 0.09 and 0.60, with the strongest agreement observed for *Trichostrongyle* eggs at T3 (CCC = 0.60; 95% CI: 0.40–0.75). Across all parasite types and sampling periods, the Parasight^®^ automated device consistently underestimated fecal egg counts compared with the manual reference method. The mean percentage bias ranged from −48.5% to −81.8%, indicating a systematic undercount by the automated system.

The 95% ratio limits of agreement (LoA) were broad, reflecting considerable variability between the two methods. For instance, at T1 for *Trichostrongyle* eggs, Bland–Altman analysis showed that Parasight^®^ readings could range from only 5% of the manual count (ratio = 0.05) to as much as 74% higher (ratio = 1.74).

Similar patterns were observed for *Strongyloides* and Total EPG, with limits spanning up to 6-fold differences at higher egg counts in T1. Agreement improved modestly at T2 and T3, with *Trichostrongyle* remaining the most consistent variable (CCC = 0.48 at T2 and 0.60 at T3), supported by relatively strong monotonic associations with the manual counts (ρ = 0.76 at T2 and 0.83 at T3). However, the broad limits of agreement across all parasites and time points indicate substantial variability between methods, suggesting that while Parasight^®^ automated device captures overall trends, its absolute agreement with manual McMaster counts remains limited. Despite poor absolute agreement, Spearman’s rank correlations were moderate to high (r = 0.52–0.83), indicating that Parasight^®^ automated device tracked the relative ranking of samples reasonably well, even though absolute values differed. Comparison between the Parasight^®^ automated device automated system and the average of two manual McMaster counts demonstrated a small but consistent negative bias, with Parasight^®^ automated device yielding slightly lower log-transformed FECs across all sampling times (Figure 2D–F).

### 3.3. Inter-Rater and Human–Machine Agreement in Categorical Classification of Trichostrongyle Egg Counts

Cohen’s Kappa (κ) was used to evaluate categorical agreement (≤1000 vs. >1000 EPG of *Trichostrongyle*) between each of the manual observers and between the average of manual observers and the automated Parasight^®^ automated device. Agreement between the two observers was consistently high across sampling times, with perfect agreement at T1 (κ = 1.00, *p* < 0.001; 100% raw agreement), almost perfect agreement at T2 (κ = 0.94, *p* < 0.001; 97.5% raw agreement), and substantial agreement at T3 (κ = 0.77, *p* < 0.001; 90.3% raw agreement).

In contrast, agreement between average of the two manual observers and the Parasight^®^ automated device classification was lower. At T1 and T2, the level of agreement was fair (κ = 0.34 and 0.38, respectively; *p* < 0.01), with raw agreements of 69.4% and 65.0%. At T3, agreement improved to a moderate level (κ = 0.54, *p* < 0.001; 77.4% raw agreement).

### 3.4. Diagnostic Performance of Parasight^®^ Automated Device

**Figure 3:**
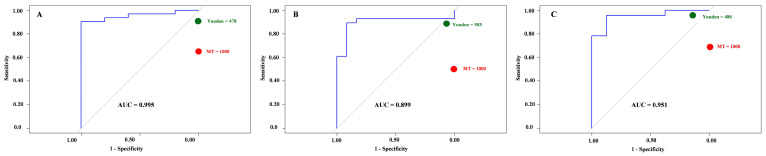
Receiver operating characteristic (ROC) curves illustrating the diagnostic performance of the Parasight^®^ automated system compared with the manual McMaster reference method across 3 sampling times (T1–T3). Panels (**A**–**C**) correspond to 1st, 2nd and 3rd sampling times which are T1, T2, and T3, respectively. Each plot shows the area under the curve (AUC) indicating overall discriminatory ability, with green points representing the optimal cutoff determined by Youden’s J statistic (478, 503, and 486 EPG for T1, T2 and T3) and red points representing the fixed manual treatment threshold (1000 EPG). The red points illustrate that increasing the threshold to 1000 EPG decreases sensitivity while maintaining the specificity.

ROC analysis was used to evaluate the ability of Parasight^®^ automated device to classify animals above the treatment threshold of >1000 EPG as defined by the manual McMaster reference. The Parasight^®^ automated device showed excellent discrimination with an AUC of 0.96. The optimal cutoff identified by Youden’s J statistic was 478 EPG, which provided 90% sensitivity, 100% specificity, and 100% positive predictive value. At this threshold, the negative predictive value was 62.5%, indicating that most positives were correctly identified, although some false negatives remained. When applying the fixed manual cutoff of 1000 EPG to the Parasight^®^ automated device counts, specificity and PPV remained 100%, but sensitivity declined to 65%, and NPV fell to 31%. This suggests that while the Parasight^®^ automated device rarely overestimates egg counts, it systematically underestimates relative to the manual McMaster, leading to missed cases when the same treatment threshold is applied. Adjusting the AI cutoff to ~480 EPG may therefore be necessary to align treatment decisions with manual results. Figure 3A illustrates the ROC plot showing the diagnostic performance of the Parasight^®^ device for detecting samples above 1000 EPG as determined by the manual McMaster method at T1. At T2, the Parasight^®^ automated device demonstrated excellent discrimination against the manual McMaster reference, with an AUC of 0.90. The optimal cutoff identified by Youden’s J statistic was 503 EPG, which provided 89% sensitivity, 92% specificity, 96% positive predictive value, and 79% negative predictive value. At this threshold, Parasight^®^ automated device achieved a balanced performance, detecting nearly all animals requiring treatment while maintaining few false positives. When the fixed clinical cutoff of 1000 EPG was applied to Parasight^®^ automated device counts, specificity and PPV remained 100%, but sensitivity declined to 50% and NPV to 46%. This indicates that although Parasight^®^ automated device never misclassified uninfected animals as requiring treatment, it missed approximately half of the truly positive cases above the manual threshold. These findings suggest that Parasight^®^ automated device systematically underestimates egg counts relative to McMaster at T2, and that adjusting the operational cutoff to ~500 EPG may better align treatment decisions with the manual gold standard. Figure 3B illustrates the ROC plot showing the diagnostic performance of the Parasight^®^ automated device for detecting samples above 1000 EPG as determined by the manual McMaster method at T2. At T3, Parasight^®^ automated device showed an AUC of 0.95. The optimal cutoff identified by Youden’s J was 486 EPG, yielding 96% sensitivity, 88% specificity, 96% PPV, and 88% NPV. This threshold provided strong overall diagnostic performance, ensuring that nearly all positive animals were detected while keeping false positives rare. When applying the fixed clinical cutoff of 1000 EPG to Parasight^®^ automated device results, specificity and PPV remained at 100%, but sensitivity declined to 70% and NPV to 53%. Thus, although Parasight^®^ automated device did not misclassify any negatives as requiring treatment, it missed approximately one-third of true positives above the manual threshold. These findings confirm a consistent tendency of Parasight^®^ automated device to underestimate egg counts relative to the manual method and highlight that a lower cutoff (around 480–500 EPG) may be more appropriate to align treatment decisions with manual McMaster results at T3. Figure 3C. illustrates the ROC plot showing the diagnostic performance of the Parasight^®^ automated device for detecting samples above 1000 EPG as determined by the manual McMaster method in the T3. 

### 3.5. Regression Analysis

**Figure 4:**
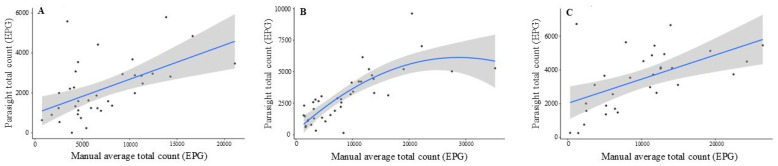
Association between manual fecal egg counts and Parasight^®^ automated egg counts across three sampling times (T1–T3). Scatterplots show the relationship between the manual average total egg count (EPG) from two observers and the Parasight^®^ total egg count at 1st sampling time (T1, panel (**A**)), 2nd sampling time (T2, panel (**B**)), and 3rd sampling time (T3, panel (**C**)). The blue line represents the fitted regression model for each timepoint, with the shaded gray region indicating the 95% confidence interval of the prediction band. At T1 (panel (**A**)), the association between methods was best described by a linear model (quadratic term non-significant), with Parasight^®^ counts increasing by approximately 0.17 EPG for every 1-EPG increase in manual counts (*p* < 0.001; R^2^ = 0.29). At T2 (panel (**B**)), a quadratic model provided the best fit (R^2^ = 0.66). At T3 (panel (**C**)), the relationship again followed a linear pattern (quadratic term non-significant), with Parasight^®^ counts increasing by approximately 0.14 EPG per 1-EPG increase in manual counts (*p* = 0.0013; R^2^ = 0.30).

At T1, the quadratic term was not significant in the full model (*p* = 0.82) and was removed. The resulting linear model, Figure 4, indicated that Parasight^®^ automated device count increased by approximately 0.17 EPG for every 1 EPG increase in the manual average (slope = 0.17 ± 0.046; *p* < 0.001). This model explained 29% of the variance in total number of EPG counted by Parasight^®^ automated device at T1 (R^2^ = 0.29). At T2, both the linear (*p* < 0.001) and quadratic (*p* < 0.001) terms were statistically significant, and the quadratic model was retained. Parasight^®^ automated device counts increased with manual counts but followed a curvilinear pattern, with a progressively flatter slope at higher egg burdens. For example, consider 1000 EPG on the manual scale, a 100-EPG increase in manual average count corresponds to an increase of roughly 40 EPG in Parasight^®^ automated device, whereas at higher counts the incremental increase per 100-EPG manual becomes smaller, indicating underestimation at the upper range. The quadratic model at T2 explained 66% of the variation in total number of EPG counted by Parasight^®^ (R^2^ = 0.66). At T3, the quadratic term again lacked significance in the full model (*p* = 0.17) and was removed. The final linear model showed that Parasight^®^ automated device counts increased by approximately 0.14 EPG for every 1 EPG increase in the manual average (slope = 0.14 ± 0.040; *p* = 0.0013). This model explained 30% of the variance in total number of EPG counted by Parasight^®^ automated device at T3 (R^2^ = 0.30).

## 4. Discussion

The present study aimed to evaluate the performance of an AI-assisted fluorescence imaging system (Parasight^®^ automated device) for fecal egg counting in Kiko goats, using the conventional McMaster method as the reference standard. As a foundational step, inter-observer reliability of the manual McMaster technique was assessed to establish a robust benchmark for comparison. Overall, the two observers demonstrated consistently high agreement across all sampling times, particularly for *Trichostrongyle* and *Moniezia* counts, confirming the reliability of the McMaster method as a gold-standard approach for fecal egg quantification. The McMaster method is currently recommended for use in veterinary practice as a simple and user-friendly method [17,18,19,20,21,22]. The Bland–Altman plots (Figure 2A–C) visually reinforced this strong concordance, showing minimal systematic bias and narrow limits of agreement between observers. These findings indicate that the manual method produces repeatable results under controlled conditions, with only minor dispersion at higher egg concentrations where counting precision typically declines. Cohen’s Kappa analysis of categorical classification (≤1000 vs. >1000 EPG) further supported this conclusion. Agreement between the two observers was perfect at T1, almost perfect at T2, and substantial at T3, with raw agreement consistently exceeding 90%. These findings confirm that both manual observers were highly consistent not only in quantitative enumeration but also in clinical classification of infection levels. Across all three sampling times, ICCs consistently reflected good agreement between observers, supporting the reproducibility of manual counting under controlled conditions. Nevertheless, Bland–Altman analysis revealed a more nuanced picture: while mean biases were modest (ranging from +749 to +1626 EPG), the limits of agreement were wide (up to −7962 to +11,106 EPG). This indicates that although observers tended to rank samples similarly, absolute discrepancies between counts could still be biologically relevant. The total EPG values can thus be viewed as an aggregate in which the variability of *Trichostrongyle* and *Strongyloides* components becomes amplified. High variability in the FECs has been reported previously (Carstensen et al., 2013) [23] and the effect of reducing sample processing time as a source of human error has been documented previously [14]. However, in this study there was no time limitation that would force the manual observer to have errors related to the shortness of time budget. Together, these results demonstrate that the McMaster method provides reliable inter-observer consistency, but its precision naturally declines as egg concentrations increase. This finding highlights the intrinsic variability of manual microscopy that must be considered when validating automated alternatives.

When comparing the Parasight^®^ automated fluorescence-imaging device with the manual McMaster method, the agreement was variable across sampling times and parasite groups. Lin’s CCC ranged from poor to moderate, indicating limited absolute agreement between the two methods. The strongest concordance was observed for *Trichostrongyle* eggs at T3, while *Strongyloides* and total EPG values showed lower levels of agreement. Despite these modest CCC values, the corresponding Spearman’s rank correlations were moderate to high, suggesting that Parasight^®^ automated device effectively captured the relative ranking of infection intensities, even when absolute counts diverged from the manual reference. A previous study [14] has shown a strong linear correlation between automated and McMaster counts (R^2^ ≈ 0.93); however, correlation primarily reflects association rather than full agreement. Similarly, earlier work by Slusarewicz et al., (2016) [15] demonstrated the feasibility of using fluorescent chitin-binding probes and smartphone-based imaging for parasite egg enumeration, showing a strong linear relationship with manual McMaster counts (R^2^ ≈ 0.93–0.98) and improved precision through automated image analysis. However, that study primarily assessed correlation and technical performance rather than full analytical agreement. In the present study, we extended this evaluation by assessing both precision and accuracy using Lin’s concordance correlation coefficient and Bland–Altman analysis

Results of the Bland–Altman analysis provided further insight into the pattern of disagreement between the two methods. Across all sampling times, the Parasight^®^ automated device consistently underestimated fecal egg counts relative to the manual McMaster technique, with mean percentage biases ranging from −48% to −82%. The 95% limits of agreement were wide, particularly at lower egg concentrations, reflecting greater variability in low-count samples. Nevertheless, most observations fell within these limits, indicating that although systematic underestimation occurred, the magnitude of disagreement remained within a diagnostically acceptable range. Similar tendencies have been reported in previous studies evaluating automated FEC technologies, where differences in flotation media, image-processing thresholds, and egg-recognition algorithms influenced count outcomes. A recent study in sheep [24] demonstrated that a fluorescence-AI platform can rapidly quantify the percentage of *Haemonchus contortus* with both strong correlation and Lin’s concordance relative to manual fluorescence microscopy and coproculture (R^2^ ≈ 0.82–0.88; qc ≈ 0.86–0.94), albeit with a subtle tendency toward lower automated percentages. These results support the broader feasibility of fluorescence-based automation. Our study complements this literature by evaluating absolute fecal egg counts in goats and by quantifying agreement with both Lin’s CCC and Bland–Altman limits. Taken together, our analyses suggest that Parasight^®^ automated device is promising, while also underscoring the value of method-specific calibration for clinical decision making.

Categorical agreement between the Parasight^®^ automated device and the average of the two manual observers was also evaluated to assess consistency in diagnostic classification. At T1 and T2, agreement was fair, with raw agreements of 69.4% and 65.0%. At T3, the level of agreement improved to moderate. These results indicate that although Parasight^®^ automated device tended to misclassify some samples near the clinical threshold, primarily underestimating those above 1000 EPG. Collectively, the continuous and categorical analyses demonstrate that the Parasight^®^ automated device reproduced overall infection trends and rankings observed with the manual McMaster method, but systematic underestimation reduced exact numerical and categorical alignment. To our knowledge, this is the first evaluation of categorical agreement (≤1000 vs. >1000 EPG) between an automated fluorescence-AI system and the manual McMaster reference. Calibration of diagnostic thresholds is therefore warranted to ensure equivalence in treatment decision-making.

Across all sampling times, ROC analysis demonstrated that the Parasight^®^ device achieved excellent overall discrimination when benchmarked against the manual McMaster reference (AUC = 0.90–0.96). Despite this strong diagnostic capacity, the device consistently underestimated egg counts, a trend also evident in Bland–Altman and concordance analyses. This systematic underestimation primarily affected samples near the clinical threshold of 1000 EPG, leading to reduced sensitivity when the manual cutoff was directly applied. At this threshold, specificity and positive predictive value remained at 100%, indicating that Parasight^®^ device rarely overestimated or falsely classified negatives as positives. However, sensitivity declined from 89 to 96% at the optimized cutoff (~480–500 EPG) to 50–70% when using the fixed manual threshold, resulting in lower negative predictive values (31–53%).

Regression analyses provided additional insight into the relationship between Parasight^®^ device and manual McMaster counts beyond agreement metrics. While concordance and Bland–Altman analyses evaluate absolute equivalence, regression characterizes how changes in manual egg counts are reflected in the automated system. At T1 and T3, the relationship between the two methods was best described by a linear model, indicating that Parasight^®^ device counts increased proportionally with manual counts, albeit at a reduced magnitude (0.14–0.17 EPG increase per 1 EPG increase manually). These shallow slopes support the consistent underestimation observed in the Bland–Altman plots and demonstrate that although Parasight^®^ device tracked directional changes in egg burden, it systematically produced lower absolute values across the observed range. In contrast, T2 exhibited a significant quadratic pattern, with a steep initial increase and progressive flattening at higher egg concentrations. This curvilinear trend likely reflects partial sensor or algorithmic saturation under high parasite loads, a phenomenon also described in prior evaluations of automated FEC platforms. The strong model fit at T2 (R^2^ = 0.66), compared with more modest fits at T1 and T3 (R^2^ ≈ 0.29–0.30), suggests that the device performed most consistently when egg counts were moderate and more variable at low or extremely high burdens. Collectively, these regression findings indicate that the Parasight^®^ device reliably captured relative differences in fecal egg shedding but exhibited non-proportional scaling and reduced accuracy as egg burdens increased, further reinforcing the need for method-specific calibration before applying manual diagnostic thresholds to automated outputs.

These findings collectively indicate that Parasight^®^ device captures the directional trend and relative infection intensity reliably, but due to undercounting bias, the same treatment threshold cannot be directly transferred from manual to automated readings. Adjusting the operational cutoff to approximately 480–500 EPG would restore diagnostic equivalence to manual classification and minimize false negatives while maintaining high specificity. Importantly, this pattern was consistent across all three sampling periods, reflecting stable device performance under field conditions. Overall, this study demonstrates that the Parasight^®^ AI-assisted fluorescence imaging system provides a robust, objective, and reproducible approach for fecal egg counting in goats, with excellent ability to rank parasite burden and discriminate animals above the clinical treatment threshold. However, systematic underestimation of egg counts relative to the manual McMaster method resulted in poor-to-moderate absolute agreement and reduced sensitivity when conventional thresholds were applied directly. These findings indicate that Parasight^®^ is best suited as a calibrated decision-support or screening tool rather than a direct numerical replacement for manual counts. Importantly, adjusting the operational cutoff restored diagnostic equivalence with manual classification, supporting its practical use in targeted selective treatment programs. From a clinical perspective, this calibration-based approach can reduce labor demands and observer variability while maintaining appropriate treatment decisions. Future research should focus on external validation across diverse production systems, assessment of within-device repeatability, and refinement of algorithmic scaling to improve absolute agreement across the full range of parasite burdens.

The findings of this study should be interpreted considering both the inherent limitations of the McMaster FEC method and those of evaluating a proprietary automated diagnostic platform. The McMaster technique has limited analytical sensitivity, does not differentiate parasite species within strongyle-type eggs, and provides a single time-point estimate that can be influenced by egg-shedding variability, animal physiology, and environmental factors [17]. Such biological and methodological variability likely contributed to part of the disagreement observed between the manual and automated methods. In addition, the Parasight^®^ system was evaluated as a fixed, commercially deployed algorithm, and its internal model architecture and training process are not publicly disclosed, limiting deeper assessment of algorithm-level sources of bias. Repeatability of the Parasight^®^ device itself was not directly evaluated in this study, as each sample was processed once to reflect routine clinical use; therefore, within-device precision could not be quantified. The consistently low Lin’s concordance correlation coefficients observed are best interpreted as reflecting systematic scale and location bias, primarily underestimation by the automated system, rather than poor association, as supported by moderate-to-high rank correlations, strong diagnostic discrimination, and coherent regression patterns. Finally, the absence of an independent external validation dataset represents an additional limitation, and future studies incorporating replicate automated measurements and multi-farm validation cohorts will be essential to fully characterize precision, robustness, and generalizability of automated fecal egg counting systems.

## 5. Conclusions

In Kiko male goats, the Parasight^®^ AI-assisted fluorescence system showed excellent ability to discriminate animals above the clinical treatment threshold (AUC 0.90–0.96) and rank parasite burden, but it had poor-to-moderate absolute agreement with the manual McMaster reference because it consistently underestimated egg counts. As a result, applying the same 1000 EPG cutoff reduced sensitivity and missed some true positives, while specificity remained high. Adjusting the Parasight^®^ operational threshold to approximately 480–500 EPG better aligned treatment classification with McMaster and supports its use in targeted selective treatment programs after calibration.

## Figures and Tables

**Table 1:** Inter-observer reliability and agreement of manual McMaster count among observers in across sampling times.

Time	Variable	Count	ICC ^3^	(95% CI)	Bias ^4^	95% LoA ^5^
T1 ^1^	*Trichostrongyle*	36	0.93	0.87–0.96	+61	−2350 to 2472
	*Moniezia*	33	0.87	0.74–0.93	−215	−1536 to 1105
	*Strongyloides*	36	0.64	0.40–0.80	+688	−3218 to 4593
	Total EPG ^2^	36	0.84	0.70–0.91	+749	−4438 to 5935
T2	*Trichostrongyle*	40	0.87	0.77–0.93	+418	−4777 to 5612
	*Moniezia*	40	0.89	0.80–0.94	−65	−683 to 553
	*Strongyloides*	40	0.86	0.72–0.93	+1209	−4265 to 6682
	Total EPG	40	0.80	0.65–0.89	+1626	−7853 to 11,106
T3	*Trichostrongyle*	31	0.85	0.72–0.93	+405	−3482 to 4292
	*Moniezia*	31	0.97	0.94–0.99	−16	−551 to 518
	*Strongyloides*	31	0.81	0.64–0.90	+589	−6730 to 7907
	Total EPG	31	0.79	0.62–0.89	+994	−7962 to 9949

^1^ First sampling time. ^2^ Egg per gram. ^3^ Intraclass Correlation Coefficient, values < 0.50 indicated poor, 0.50–0.75 moderate, 0.75–0.90 good, and >0.90 excellent reliability. ^4^ Mean difference in counts of fecal egg counts per gram of samples between two observers. ^5^ 95% limits of agreement.

**Table 2:** Concordance, bias, and limits of agreement between Parasight^®^ and manual McMaster counts across sampling times.

Time	Variable	Count	CCC ^2^	(95% CI) ^3^	Bias% ^4^	95% LoA ^5^	*ρ* ^6^
T1	*Trichostrongyle*	36	0.285	0.20–0.54	−2636.7	−7622.5 to 2349.1	0.76
	*Strongyloides*	36	0.094	−0.05–0.23	−2335.6	−6511.4 to 1840.2	0.26
	Total EPG ^1^	36	0.149	0.03–0.32	−4972.3	−12,698.1 to 2753.4	0.52
T2	*Trichostrongyle*	40	0.338	0.29–0.63	−2449.8	−10,241.2 to 5341.6	0.83
	*Strongyloides*	40	0.331	0.23–0.61	−3593.2	−11,773.3 to 4586.9	0.64
	Total EPG	40	0.235	0.19–0.52	−6043.0	−18,270.3 to 6184.4	0.82
T3	*Trichostrongyle*	31	0.369	0.40–0.75	−2023.4	−7300.4 to 3253.6	0.76
	*Strongyloides*	31	0.175	0.13–0.59	−4428.7	−14,392.9 to 5535.5	0.60
	Total EPG	31	0.145	0.25–0.62	−6452.1	−18,201.5 to 5297.3	0.59

^1^ EPG: Eggs per gram of feces. ^2^ Lin’s concordance correlation coefficient: values range from −1 to +1; values closer to +1 represent stronger agreement. Values < 0.50 indicate poor concordance. ^3^ 95% CI: 95% confidence interval for the CCC. ^4^ Bias: Mean difference in fecal egg counts per gram (Parasight^®^—manual McMaster). Negative values indicate systematic underestimation by Parasight^®^. ^5^ 95% LoA: 95% limits of agreement for the absolute difference in EPG on the original scale. ^6^ *ρ*: Spearman correlation coefficient.

## Data Availability

The original contributions presented in this study are included in the article. Further inquiries can be directed to the corresponding author.
